# Blood glucose monitoring in type 2 diabetes – Nepalese patients’ opinions and experiences

**DOI:** 10.1080/16549716.2017.1322400

**Published:** 2017-06-06

**Authors:** Sujata Sapkota, Jo-anne E Brien, Parisa Aslani

**Affiliations:** ^a^ Faculty of Pharmacy, The University of Sydney, Sydney, NSW, Australia; ^b^ St. Vincent’s Hospital Clinical School, UNSW, Sydney, NSW, Australia

**Keywords:** Self-monitoring, laboratory monitoring, qualitative research, Nepalese

## Abstract

**Background**: Blood glucose monitoring forms a vital component of diabetes care. Monitoring conducted at home using glucometers, and in laboratories by professionals, are two common methods of blood glucose monitoring in clinical practice.

**Objective**: To investigate Nepalese patients’ perceptions and practices of blood glucose monitoring in diabetes.

**Methods**: In-depth interviews were conducted with 48 Nepalese participants with type 2 diabetes in Sydney and Kathmandu. The interviews were audio-recorded, transcribed verbatim and thematically analysed.

**Results**: In Australia, most participants perceived home monitoring as useful; and both home and laboratory monitoring were conducted at fairly regular intervals. In Nepal, only a small number conducted home monitoring and the laboratory method formed the primary method of day-to-day monitoring. The laboratory method was preferred due to easy access to laboratories, lack of faith in glucometers and perceptions that home monitoring is costlier. However, overall monitoring was irregular in Nepal. In addition to the healthcare system which enabled cheaper self-monitoring in Australia, Nepalese in Australia also tended to have a better understanding about the purpose of home monitoring.

**Conclusions**: This study has highlighted the disparity in perceptions and practices related to blood glucose monitoring. Understanding the importance of blood glucose monitoring and access to affordable resources are critical facilitators for conducting regular monitoring. Both patient and health-system factors play a key role in ensuring continued diabetes monitoring and management.

## Background

The diagnosis of diabetes is by assessment of plasma glucose concentration alone, or in combination with symptoms of hyperglycaemia, such as polyuria and polydipsia [1,2].

It is known that sustained hyperglycaemia can lead to micro- and macro-vascular complications [3]. Similarly, hypoglycaemia may be life-threatening [4,5]. While hyperglycaemia is the characteristic feature of diabetes [3], patients with diabetes may also be susceptible to hypoglycaemia due to the nature of the disease [4] and treatment [6]. Maintenance of glycaemic levels within a target range is a major goal of diabetes management [7].

The use of portable glucometers by patients at home, along with laboratory tests conducted by health professionals [8], remain the two most common methods for blood glucose monitoring in patients with type 2 diabetes (T2D). While regular self-monitoring of blood glucose (SMBG) levels using glucometers is regarded as an essential tool for self-care [2,9–11], tests performed in accredited laboratories are preferred for confirming diagnoses, and are used in clinical and therapeutic decision making [2].

Self-monitoring may generate awareness of a patient’s own disease management [12]. However, effective diabetes management through SMBG may be limited by a patient’s ability to interpret and act on the ‘abnormal’ home blood glucose readings [13], by the analytical quality of the instruments, and by a patient’s knowledge about the device and their understanding of its purpose [14]. Studies report that while some patients find self-monitoring useful, it can also cause discomfort and stress, particularly when readings are ‘high’ [12,13]. In 2010, a Cochrane review concluded that SMBG was of limited clinical usefulness and unlikely to be cost effective in patients with T2D managed on oral agents or lifestyle alone [15].

Diabetes prevalence in Nepal has increased over the past three decades [16]; and there appear to be a significant number of undiagnosed cases [17]. Effective diabetes management in Nepal, including diagnosis and treatment, is challenged by multiple factors, including poor overall understanding of diabetes in the community [18], limited healthcare resources, and substandard diagnostic and laboratory facilities [19,20]. Whereas laboratory blood glucose monitoring in Nepal is accessible by patients with diabetes without the need to obtain referrals from physicians, the impact of this accessibility on rates of diagnosis and overall diabetes monitoring and management is unknown.

Understanding blood glucose monitoring practices is key to a comprehensive understanding of diabetes management among Nepalese individuals. This study, therefore, aimed to investigate Nepalese patients’ perceptions of blood glucose monitoring in diabetes, and explore their monitoring practices. Nepalese participants living in Australia and in Nepal were included in the study, which enabled an assessment of the similarities and differences in perspectives and behaviour amongst Nepalese in the two countries. This, in turn, has allowed a deeper evaluation of the factors influencing patients’ monitoring practices, including the potential impact of the healthcare system from patients’ perspectives.

## Methods

### Participants

Nepalese adults (≥ 18 years) with T2D, who were on at least one anti-diabetic medication were eligible for recruitment. Participants were recruited using several strategies (Table 1).

**Table 1.** Recruitment strategies.

Recruitment in Sydney, Australia	Recruitment in Kathmandu, Nepal
Advertisement in print (Nepalese papers) Advertisement on Facebook and Gumtree Advertisements placed in selected Nepalese grocery shops and restaurants Word of mouth Snowballing technique	Advertisement in pharmacies and medical centres within travelling distance of Kathmandu Word of mouth Snowballing technique

For participants in Australia, Nepalese origin was defined as any person who was born in Nepal or one or both of whose parents were born in Nepal, and was residing in Australia at the time of interview. The participants could be in Australia on a temporary or a permanent basis.

### Data collection and analysis

In-depth, face to face, semi-structured interviews were conducted at a time and public venue convenient for the participants, in Sydney and in Kathmandu. The interviews were based on a protocol which was designed to address broader research aims, and was divided into nine domains of inquiry (Table 2).

**Table 2.** Broad issues addressed by the interview protocol.

	Issues explored by the study
1.	Perceptions of being a diabetic
2.	Overall diabetes management*
3.	Perceptions and beliefs about medications for diabetes
4.	Information and information sources*
5.	Perceptions about own knowledge and understanding
6.	Medication adherence
7.	Support in diabetes management and medication taking
8.	Perceptions of strategies to address overall diabetes management and medication taking
9.	Perceptions of impact of Nepalese culture on diabetes and medication taking

*Findings related to monitoring practices and behaviours emerged in these domains.

The protocol was first tested for face and content validity [21] with a sample of four participants (not included in the study) in Nepal, by the first author. No changes were made as a result of the testing. Interviews were conducted in both countries until repetition and redundancy in the data (that is, until data saturation) [22] were evident. All interviews were conducted by the first author in Nepali language and were audio-recorded. The average duration of the interviews was approximately one hour (range 0.6–2.3 hours).

All interviews were transcribed verbatim for thematic analysis [23]. The first three transcripts were translated into English and independently analysed by two researchers (first and third authors). Findings relevant to the study aims were coded from as many perspectives as possible; that is, open coding using an inductive approach was applied [24]. The initial codes were categorised, organised or grouped into themes and sub-themes [23,24]. Consensus was reached regarding the themes, and the remaining interviews were analysed by the first author. Themes were refined continuously through regular research team discussions. A constant comparison approach [25] was used throughout data analysis. The interviews from the two cohorts were separately analysed. The findings were compared between the two cohorts to determine differences and similarities. Only the findings related to diabetes monitoring practices (an emergent theme) have been reported. Findings related to participants’ perceptions and behaviours regarding diet [26] and medication taking [27,28] have been reported elsewhere.

## Results

### Participant demographics

Eighteen participants were interviewed in Australia and 30 in Nepal (Table 3). Of the 18 in Australia, 12 were residing in Sydney on a permanent basis and six were parents visiting their children who lived in Sydney. Those permanently residing in Sydney were all first-generation migrants.

**Table 3.** Participants’ demographics.

Characteristics	Sydney n (%)	Kathmandu n (%)	All participants n (%)
**Gender**			
Male	12 (66.7)	18 (60)	30 (62.5)
**Age (years)**			
Median Range	54.2 24.0–73.0	54.5 33.0–80.0	55.5 24.0–80.0
**Duration of diagnosis**			
Median Range	8.2 years 8 months–20 years	9.0 years 1 month–30 years	7.7 years 1 month–30 years

### Blood glucose monitoring practices

Study participants reported that they measured their blood glucose for a number of reasons: as a routine general healthcare measure to keep track of their diabetes, because of advice from healthcare professionals, or when they experienced symptoms that made them feel that their ‘blood sugar’ was not under control. A few participants also measured to observe and understand the effects of food, exercise or medications on their blood glucose levels. While participants in Nepal monitored both fasting and postprandial blood glucose levels, participants in Australia mostly monitored fasting levels. Laboratory-based monitoring was utilised by all participants; however, routine use of glucometers for self-monitoring was more common in participants residing in Australia. The monitoring practices of those participants visiting Australia changed when they were in Australia compared to when in Nepal. Although they conducted home-based monitoring while in Australia, they preferred laboratory monitoring in Nepal.

### Home blood glucose monitoring

Home blood glucose monitoring (HBGM) was more frequently conducted by participants interviewed in Australia. This difference appeared to be facilitated by their reported better understanding of the purpose of HBGM, and the availability of free or subsidised equipment (glucometers and strips). There appeared to be differences in perceptions and attitudes towards self-monitoring between the two cohorts.

#### HBGM practices

Of the 12 participants residing in Australia, eight were conducting HBGM regularly; the frequency of measurement varied from twice daily to once a week (Table 4, Q1). Some reported that they would monitor several times a day if they found that their blood glucose level had increased (Table 4, Q2), with one reporting that he used his device only when he felt ‘uncomfortable’ (A05). Two participants had also maintained a ‘log-book’ of their HBGM results to track their glycaemic control. Another two stated that although they had used glucometers regularly initially for about a year, they had discontinued this, despite having a device at home (Table 4, Q3).

**Table 4.** Quotes about HBGM perceptions and practice among participants in Australia.

HBGM: Nepalese in Australia	
**Q1**	*Monitoring and adjusting lifestyle based on results* ‘I [check] almost everyday [with] that sugar test machine that I have? In a week almost five to six times, I am doing that [checking the blood sugar]. When I do that [the glucose level] it almost always stays at six on an average. Sometimes less than six. And if sometimes, if it is increased, what I do is, I control my food. I don’t eat carelessly.’ (A14)
**Q2**	*Frequency of monitoring is dependent on the results* ‘Last night, there was a party, the next day after that when I checked my blood sugar level, it had gone very high; when I saw that, that day, I again checked it three to four times in that day. So in such circumstances when you suddenly see an unexpected increase, I check around three to four times a day as well.’ (A01)
**Q3**	*Regular monitoring can be stressful* ‘Initially I used to check regularly, almost every day. Initially after diagnosis straightaway I bought the machine and started [testing], after I got the results of the GTT [Glucose Tolerance Test] test, with doctor’s recommendation. After almost one year, the doctor told that it is not necessary to test every day. This is not necessary; it’s a waste of money. That will for no reason increase the stress! “Why has it increased today, why [has it] decreased?” you keep on thinking about that. That’s why it is not necessary. The doctor told that, which you are doing every three month [in laboratory] is enough. In between if you feel like you can check once or twice. So, I stopped.’ (A10)

Only four participants out of 30 interviewed in Nepal reported that they were conducting HBGM and three of these individuals were using insulin. A further six participants mentioned that they had a device at home, but did not use it; and five reported that they had used a device or had owned one in the past. Nine stated they had heard about a device but had never used one, and the remaining few did not appear to be aware of HBGM. Six out of 30 participants reported that the glucometers had been sent to them by their children/relatives from abroad.

#### Perceptions and factors impacting HBGM

Most participants in Australia perceived HBGM as highly useful in keeping track of their glycaemic control, for detecting unexpected rises or falls in glycaemic levels, as well as for understanding influences on their blood glucose levels such as the effect of different types/amounts of food or the effect of exercise. They reported that adjusting their lifestyle based on the results of HBGM helped them to better manage their glycaemic levels. However, daily monitoring was reported as stressful by a few, leading to reduced frequency of use.

The participants in Australia reported that the need to conduct HBGM was reinforced by their doctors. Furthermore, conducting HBGM was reported to be affordable for people residing permanently in Australia, due to the services/facilities they received as members of the Australian Diabetes Association (ADA). Glucometers were provided free of charge, and the strips were available at subsidised rates. They felt that had similar facilities been available in Nepal, this would promote self-monitoring and better diabetes management.

In Nepal, except a few (n = 5) who considered HBGM as a quick, efficient and easy way to monitor blood glucose levels, most had doubts about the reliability of the instruments (Table 5, Q4) or did not appreciate a need for having a glucometer at home. Many participants reported receiving information about the unreliability of HBGM from friends, relatives or other people with diabetes. They tended to compare laboratory reports with glucometer readings to assess the glucometer’s reliability.

**Table 5.** Quotes about HBGM perceptions and practice among participants in Nepal.

HBGM: Nepalese in Nepal	
**Q4**	*Unreliable/not accurate* ‘Yes, I checked [using glucometer]. [The machine] that [I found] was not accurate. It was wrong (laughs). The one that we go and checked in the laboratory? We go there. The [reading from] the machine is not that clear. I bought that spending almost 3000–3500 rupees. Then when I checked, if it [blood sugar] was 160 [mg/dl], the number in my body, in that [glucometer] it showed 350, 380! That machine with the battery. Then I did not have faith in that.’ (N24)
**Q5**	*Laboratory monitoring is more affordable* ‘Now to test the blood sugar [in lab] they take 100 rupees, [both] fasting and PP [postprandial]. They take 100 rupees. Now tell me, is it easy to pay 100 rupees or to bring that machine spending 5000–7000 [rupees] to find out that your blood sugar has increased, and that you have go to the doctor. So, that is useless.’ (N16)
**Q6**	*Stressful and painful* ‘While I was in America, my son used to prick me three times every day. You know that [glucometer] was available there? My son bought, and used to prick me three times! [That caused] more stress [concern], [blood sugar] increased more and more! [After coming back] oh… I distributed that [glucometer]. I gave it away, did not use it. And I was scared to prick as well! That you had to prick in your finger, I was scared! No I don’t use it [here]!’ (N27)
**Q7**	*Not essential/not necessary* ‘Look I would like to have a machine, which will tell me that you have this and this problem, and you have to take so and so medications after checking my blood sugar. I need that type of machine. The machine has to tell that. Now it tells you that your blood sugar is this, but then you have to go to the doctor again [what’s the point?]’ (N16)

Furthermore, participants in Nepal reported that in general, home monitoring was more expensive than laboratory monitoring (Table 5, Q5), more stressful and painful (Table 5, Q6), and more essential for people with unstable/fluctuating blood glucose levels. A few also felt that keeping a glucometer was futile, given that they would eventually require laboratory reports (for their doctor) and/or a visit to a doctor to seek advice for management in case the results were not within the ‘normal range’ (Table 5, Q5, Q7). An elderly female reported she did not use the glucometer at home, as she did not ‘really know how to use the blood sugar (device)… had tried once or twice, but left it as is after that’ (N08).

### Laboratory-based blood glucose monitoring

Overall, laboratory-based monitoring was understood as a more reliable method of testing blood glucose than using glucometers (by participants in both countries).

#### Laboratory monitoring practices

Participants residing in Australia described a consistent pattern of laboratory monitoring. Based on referrals from doctors, participants generally undertook laboratory tests every three to six months, or at shorter intervals where necessary with regular follow-up consultations with their doctors. Moreover, laboratory testing involved a ‘comprehensive review’ where, in addition to blood glucose, HbA1c, urea, creatinine and lipid profiles were also tested routinely.

In Nepal, the frequency of laboratory monitoring overall was higher than for participants in Australia, albeit highly inconsistent (Table 6, Q8). Just over one third of the participants reported that they monitored their blood glucose once every month or every two months. A few reported that they conducted the monitoring only before scheduled doctor appointments, and others only following symptoms or physical unwellness (Table 6, Q8–Q9). In some, laboratory monitoring was more consistent and regular during the initial days of diagnosis and gradually declined over time.

**Table 6.** Quotes about laboratory-based monitoring among patients in Nepal and in Australia.

Laboratory monitoring: Nepalese in Nepal	
**Q8**	*Inconsistent pattern of monitoring* ‘Blood monitoring, sometimes I do in 3 months, sometimes in 2 months, sometimes in 15 days, sometimes even in a week. And about why like that, if my body symptoms is telling me, diabetes is little increased, [I feel] if it’s increased, like let’s say, my body feels a little heavy, if somewhere there is pain… Now people with diabetes are hungry more often, isn’t it? Or let’s say if I feel that there is something wrong with my body, and I feel curious if the diabetes has increased, then I go and get it [blood sugar] checked.’ (N02)
**Q9**	*Perception/attitude and influencers of monitoring* ‘It’s been quite a while that I haven’t checked [the blood sugar]. [I] had checked last Magh [around December, about eight to nine months before]. And I haven’t got it checked since! No more than once a year! When I am controlling, I know what has happened to me. Now if sugar is increased, the frequency of urination will be more, I will be more hungry. And if it is decreased, I wouldn’t be able to walk due to vertigo. Then, I will know by myself! Why should I monitor more? And feed them [the laboratory] more money?’ (N09)
Laboratory monitoring: Nepalese in Australia	
**Q10**	‘The blood test here, blood test is free. Free, everything is free! The medical everything, we don’t have to pay, not even [for] the GP visit. Now because we are diabetic patients, we have the facilities to do blood test every three months, everything. We do everything, blood sugar, urea, creatinine, everything! Because of the facilities we have encouragement here. People in Nepal hesitate to pay, yes or no? And it’s expensive there too.’ (A14)

Only a couple of participants in Nepal explicitly stated that they regularly monitored HbA1c and appeared to be aware of the importance of HbA1c measurement. Most had little understanding about the tests that they were recommended, except for fasting and postprandial blood glucose levels. A few participants who had brought their files or recent prescriptions to the interview did have records of HbA1c tests. However, it was unclear if participants in Nepal routinely tested for HbA1c.

#### Factors impacting laboratory monitoring practices

Overall, financial issues, poor time management and limited understanding about diabetes were reported to have had an impact on participants’ laboratory monitoring practices. Participants in Australia mentioned that blood glucose testing was free for them, and consequently monitoring was easy and affordable (Table 6, Q10). However, in Nepal, while some participants reported that the doctors told them to routinely conduct laboratory tests, others reported that information regarding monitoring from doctors was restricted to getting their blood glucose measured and the report brought to their next visit – and/or measuring their blood glucose levels if they felt something was wrong.

The method of monitoring used by the participants visiting their children in Sydney varied based on whether they were in Nepal or in Australia. Whilst they would use HBGM in Australia, they preferred laboratory testing in Nepal. Five of six participants visiting Australia had an HBGM device, which they used for monitoring while in Australia. The frequency of monitoring was variable and in most cases did not follow a specific pattern. Only one participant reported regular monitoring with a glucometer while in Nepal. In Australia, they reported that laboratory monitoring was ‘bothersome’ (A02) and as visitors, felt unfamiliar with the systems and surroundings. Furthermore, due to the lack of healthcare entitlement as visitors, the monitoring was also reported as costlier than in Nepal.

## Discussion

We investigated Nepalese participants’ perceptions of blood glucose monitoring practices, their monitoring behaviour and explored the differences in practice and perceptions between Nepalese living in Nepal and Australia. Overall, participants in Australia were more regularly monitoring their blood glucose levels than those in Nepal. While most participants in Australia conducted home monitoring, laboratory monitoring was also conducted. In Nepal, participants mostly sought laboratory monitoring, albeit irregularly, and few practised home monitoring.

Laboratory monitoring in Nepal was performed not only by the doctor for periodic assessment of the patients’ diabetes control, but also by patients for routine assessment. A number of factors contributed to the popularity of laboratory monitoring in Nepal, for example, easy access to laboratories, doubts associated with glucometer readings and perceived higher cost of self-monitoring.

In contrast to the situation in Australia, where laboratory monitoring requires a doctor’s referral, in Nepal laboratory testing is directly available to patients. In Nepal, patients would seek a laboratory test either upon a recommendation from a doctor, or whenever they felt a test was required. Once they obtained the laboratory result, patients tended to make lifestyle (or therapy) adjustments on their own without necessarily consulting with a doctor [27,28]. It has been previously reported that a few of these patients delayed professional consultation after finding out about their increased blood glucose level from tests conducted in laboratories in order to avoid being initiated on medications [28]. They were also likely to alter the dose of their current medications based on blood glucose reports [28].

Participants doubted the reliability of the glucometers, possibly because of their limited understanding of the devices. This lack of trust in glucometer reliability, together with the reported observation that doctors wanted laboratory tests to make decisions about therapies and interventions, appeared to convince participants that laboratory blood glucose testing was superior to the HBGM devices [14]. Additionally, the limitations of participants’ own abilities to use glucometers or the perceived (lack of) usefulness of glucometer readings may have influenced their behaviours.

A further factor contributing to the higher use of laboratory monitoring in Nepal was the perceived lower cost of laboratory monitoring compared to home monitoring. In Nepal, participants reported having to pay for their glucometers and strips, whilst in Australia, glucometers were reported to have been provided for free with subsidised test-strips.

The reduced use of glucometers may also be due to limited participant understanding of the importance and purpose of self-monitoring. Some participants in Australia reported using HBGM to not only monitor their diabetes, but also to understand the impact of diet and exercise on their blood glucose levels. However, in Nepal, the idea that HBGM could facilitate patient empowerment and self-management [2,29] appeared poorly understood.

An additional factor influencing lower HBGM use compared to laboratory testing, as reported by the participants, may be some participants’ inability to use HBGM, and/or pain and discomfort associated with daily finger pricking. Laboratory monitoring may have been considered favourably or perceived as more practical, as the blood is taken by someone else and this was less frequent than HBGM.

Studies assessing monitoring in patients with diabetes chiefly focus on self-monitoring. No studies assessing patients’ laboratory monitoring practices were identified, presenting a gap in the literature. This study underlines the importance of patients’ understanding and beliefs about laboratory monitoring practices. This may be particularly important in settings where patients can easily access laboratory tests, and believe that these tests are more reliable and easier to undertake than self-monitoring. Patients’ perceptions and practices around laboratory monitoring could influence their perceptions and utilisation of HBGM, and overall diabetes management.

In Nepal, access to laboratory services without a doctor’s order offers patients a choice of going to (and paying for) laboratory testing, or conducting HBGM. Participants in this study therefore evaluated the pros and cons of each method to make a choice, particularly in deciding whether to conduct HBGM. Whilst laboratory monitoring offered readings that were more ‘trustworthy’ for interventions by doctors, HBGM did help the patients in tracking the everyday impact of lifestyle (diet and exercise) and offered opportunities for timely action. Educating patients on how each can be valuable can enable them in making a choice based on a sound assessment. The higher frequency of laboratory monitoring practices reported will have implications of increased costs for the healthcare system in Nepal. However, this higher frequency may also be viewed positively as it demonstrates a more stringent approach to blood glucose monitoring adopted by the patients in Nepal. Whilst these implications need further investigation, in Nepal, it is equally important to ensure that laboratory services offered are of good quality [30] and that qualified health professionals capable of providing adequate consultation and/or referrals based on the reports are in place.

There may be lessons to be learned from Australia, in that provision of cheaper home monitoring by offering free glucometers and subsidised test-strips, with healthcare professional reinforcement and education about self-monitoring practices, may be possible solutions to improve self-monitoring practices in Nepal. Nonetheless, the impact and the feasibility of transferring these services and strategies to a different context and a vastly different healthcare system, such as that of Nepal, need careful consideration [15]. Although provisions for free glucometers and subsidised test-strips may be cost-effective to patients, this could have an impact on the already limited healthcare budget in Nepal [31]. The clinical benefits of investing in self-monitoring in patients with T2D who are not treated with insulin, especially when funds are limited, have not been proven [15,32]. Furthermore, whilst guidelines recommend regular self-monitoring for all insulin-treated patients [2], no specific guidelines exist for patients with T2D on oral agents or on lifestyle strategies alone. Also important is to consider that home monitoring is effective only when patients are able to interpret and act on results [33], and if patients are able to self-adjust therapy [15].

In a country like Nepal, where diabetes imposes a huge financial burden on patients [34], and where diabetes management is challenged by poor health literacy [35] and patients’ limited knowledge of diabetes [18,36], the extent to which self-monitoring should be promoted and to what extent the benefits of self-monitoring may be achieved (and how) should be carefully considered. It is also important to have a realistic expectation of how self-monitoring practices might be implemented effectively. In Nepal, it may also be important to consider if, and how much responsibility patients are willing to accept in managing their diabetes. Whilst self-monitoring can enhance patients’ self-management skills [12,13] and may have favourable outcomes, it can impose a burden affecting patients’ quality of life [12,13]. Before making recommendations for the health system to include a subsidised self-monitoring programme in Nepal, it is also imperative to explore self-monitoring practices in patients for whom self-monitoring is considered more useful, that is, patients with type 1 diabetes and those with T2D on insulin [2].

## Limitations

This study is not able to report on patients’ actual diabetes control, and whether participants in Australia had better diabetes management. Moreover, as all interviews were conducted in Kathmandu, the healthcare hub of the country, the study is unable to comment specifically on the monitoring practices in rural Nepal, where healthcare resources are even more limited.

Future research should assess the impact of patients’ perceptions about monitoring on their actual monitoring practices and on their diabetes control. In Nepal, research should also consider how patients in rural settings monitor their blood glucose levels, and their perceptions about monitoring.

Participants’ demographic characteristics, such as socio-economic status and literacy or education level, were not assessed. The association of these features with participants’ blood glucose monitoring practices, therefore, could not be investigated and should be the focus of future research.

## Conclusions

Laboratory monitoring formed the major method of monitoring blood glucose levels by participants with T2D in Nepal. Participants’ preference for laboratory monitoring over home monitoring in Nepal was related to the direct and easy access to laboratories, participants’ perceptions that laboratory monitoring is cheaper compared to home monitoring and their inadequate understanding of the purpose of home monitoring. Whilst home monitoring was appreciated and used by participants in Australia, a very small number of participants in Nepal conducted home monitoring.

Educating patients about the importance of timely monitoring, and in the value of both self- and laboratory monitoring methods (while in parallel improving the quality of laboratory services) will promote effective diabetes monitoring in Nepal. Recommending a policy of routine self-monitoring should be based on a sound assessment of the financial implications, as well as patients’ need, preference and ability to self-monitor and to self-manage their diabetes.

## Supplementary Material

Supplementary file

## Paper context

Blood glucose monitoring is an important aspect of diabetes management. Studies conducted in patients with T2D primarily report patients’ self-monitoring practices; patients’ laboratory monitoring practices are not reported. Nepalese patients’ monitoring practices and their perceptions about blood glucose monitoring have not been explored previously.
