# Supplementary material for: Blood glucose monitoring in type 2 diabetes – Nepalese patients’ opinions and experiences
Source: Glob Health Action. 2017 Jun 6;10(1):1322400. doi: 10.1080/16549716.2017.1322400 (PMC5496077; doi:10.1080/16549716.2017.1322400)
Supplement: Supplementary file [file ZGHA_A_1322400_SM4899.docx]

Consolidated criteria for reporting a qualitative study (COREQ) for the above study

| **Personal characteristics** | | |
| --- | --- | --- |
| 1. | Interviewer/ facilitator | Sujata Sapkota (SS) |
| 2. | Credentials | BPharm, MPharm (Pharmaceutical care) |
| 3. | Occupation | PhD Candidate and registered pharmacist |
| 4. | Gender | Female |
| 5. | Experience and training | A registered pharmacist in Nepal; with experience in academia. Trained in qualitative research. |
| **Relationship with participants** | | |
| 6. | Relationship established | Prior to the commencement of each interview, the interviewer, to the best of her ability, established rapport with the participant. |
| 7. | Participant knowledge of the interviewer | Each participant received a Participant Information Statement, as required and approved by the Human Research Ethics Committee of The University of Sydney, Australia, and the Nepal Health Research Council which outlined the project aims. The researchers’ name and affiliation with The University of Sydney was also included as part of the letter head that appeared on the document. Thus, participants had knowledge of this.  The interviewer also introduced herself to the participants (name, affiliation, and role in the research) prior to the commencement of each interview. |
| 8. | Interviewer characteristics | PhD student, researcher and pharmacist from Nepal. To the best of our knowledge, the interviewer remained unbiased throughout the discussions. |
| **Theoretical framework** | | |
| 9. | Methodological orientation and Theory | No methodological orientation underpinned the study design. Thematic analysis was used to analyse the findings |
| **Participant selection** | | |
| 10. | Sampling | Convenience sampling through snowballing technique was used. Participants were selected based on set inclusion criteria- adult Nepalese patients with Type 2 diabetes on at least one medication were recruited. |
| 11. | Method of approach | Various methods were used for recruitment. These have been specified in methods section in the text (page 4, Table 1). |
| 12. | Sample size | 48 participants completed the study |
| 13. | Non-participation | 10 (reasons not enquired) |
| **Setting** | | |
| 14. | Setting of data collection | Public venue suitable for the participant |
| 15. | Presence of non-participants | In 2 interviews, the participants were accompanied by their spouses. In all others, only the participant and the researcher were present. |
| 16. | Description of sample | Nepalese ethnicity.  Basic demographic data in relation to the sample has been provided (Results section, Table 3, Page 6). |
| **Data Collection** | | |
| 17. | Interview guide | The major topics addressed by the interview guide are outlined Table 2. |
| 18. | Repeat interviews | Repeated interviews were not carried out and are not applicable to this study. |
| 19. | Audio/visual recording | Audio recording was used to collect the data. |
| 20. | Field notes | Field notes were made immediately after each interview. |
| 21. | Duration | The duration of the interviews was approximately 1 hour |
| 22. | Data saturation | Data saturation was reached. |
| 23. | Transcripts returned | Transcripts were not returned to participants for comment or correction. Transcripts were transcribed verbatim and checked against the audio to ensure accuracy prior to analysis. |
| **Data analysis** | | |
| 24. | Number of data coders | First 3 transcripts were translated into English and independently analysed by 2 researchers (SS & PA); and the remaining analysed by 1(SS). |
| 25. | Description of the coding tree | A coding tree has not been described. This manuscript details the findings of one single theme, Blood Glucose Monitoring, which emerged from the interviews. |
| 26. | Derivation of themes | Broad themes were used to develop the interview guide, in order to address the study objectives. Additional themes emerged from the interviews, one of which is presented here. |
| 27. | Software | Microsoft Word was used to manage the data. |
| 28. | Participant checking | Participants did not provide feedback on the findings. |
| **Reporting** | | |
| 29. | Quotations presented | Representative quotations have been presented in Tables 4 to 6. |
| 30. | Data and findings consistent | There is consistency in the data and findings presented. |
| 31. | Clarity of major themes | Major theme, applicable to this manuscript, is presented. |
| 32. | Clarity of minor themes | Minor themes, applicable to this manuscript, is presented. |
